# Identification and validation of a five-gene prognostic signature based on bioinformatics analyses in breast cancer

**DOI:** 10.1016/j.heliyon.2023.e13185

**Published:** 2023-01-27

**Authors:** Xin-jie Du, Xian-rong Yang, Qi-cai Wang, Guo-liang Lin, Peng-fei Li, Wei-feng Zhang

**Affiliations:** a Department of Thyroid and Breast Surgery, LongYan First Hospital, Longyan, 364000, Fujian, China; b Department of General Surgery, Linhai Hospital of Traditional Chinese Medicine, Linhai, 317000, Zhejiang, China

**Keywords:** Breast cancer, Prognostic signature, Bioinformatics analysis

## Abstract

**Background:**

This study aimed to identify prognostic signatures to predict the prognosis of breast cancer (BRCA) patients based on a series of comprehensive analyses of gene expression data.

**Methods:**

The RNA-sequencing expression data and corresponding BRCA patient clinical data were collected from the Cancer Genome Atlas (TCGA) and the Gene Expression Omnibus (GEO) datasets. Firstly, the differently expressed genes (DEGs) related to prognosis between tumor tissues and normal tissues were ascertained by performing R package “limma”. Secondly, the DEGs were used to construct a polygenic risk scoring model by the weighted gene co-expression network analysis (WGCNA) and the least absolute shrinkage and selection operator Cox regression (Lasso-cox) analysis method. Thirdly, survival analysis was performed to investigate the risk score values in the TCGA cohort. And the enrichment analysis, immune cell infiltration levels analysis, and protein-protein internet (PPI) analysis were performed. Simultaneously, the GEO cohort was used to validate the model. Lastly, we constructed a nomogram to explore the influence of polygenic risk score and other clinical factors on the survival probability of patients with BRCA.

**Results:**

A total of 1000 DEGs including 396 upregulated genes and 604 downregulated genes were identified from the TCGA-BRCA dataset. We obtained 5 prognosis-related genes, as the key biomarkers by Lasso-cox analysis (*FBXL19*, *HAGHL*, *PHKG2*, *PKMYT1*, and *TXNDC17*), all of which were significantly upregulated in breast tumors. The prognostic prediction of the 5 genes model was great in training and validation cohorts. Moreover, the high-risk group had a poorer prognosis. The Cox regression analysis showed that the comprehensive risk score for 5 genes was an independent prognosis factor.

**Conclusion:**

The 5 genes risk model constructed in this study had an independent predictive ability to distinguish patients with a high risk of death from those with a low-risk score, and it can be used as a practical and reliable prognostic tool for BRCA.

## 1 Introduction

Breast cancer (BRCA), a heterogeneous disease with a high level of morbidity, is the second leading cause of cancer mortality in women [1]. Moreover, its incidence continued to grow globally and surpassed lung cancer to become the top one frequently diagnosed cancer worldwide in 2020 [2]. In addition, BRCA patients have poorer quality of life and shorter survival due to their higher mortality rate and the character of intratumor heterogeneity. Even so, the clinical treatment plan and prognosis judgment are made according to the TNM grade of the tumor, the clinical stage of the tumor, the level of hormone expression, and the expression level of *HER2* because of the lack of reliable prognostic biomarkers. It is urgent to identify novel biomarkers to improve the current prognosis and possible treatment strategy of proliferation and metastasis in BRCA research to further improve disease outcomes.

Precision medicine, a new medical model, pursues the correct selection and precise application of appropriate diagnosis and treatment methods for each patient using gene sequencing, bioinformatics, and big data, to minimize iatrogenic damage, minimize medical costs, and maximize patient benefits [3]. Consequently, the mining of the global co-expression network modules and the identified intracellular hub genes are quite important for the upcoming precise diagnosis and treatment of BRCA. There are some related researches as follows. Wang et al. study indicated that nine ferroptosis-related genes (*ALOX15*, *CISD1*, *CS*, *GCLC*, *GPX4*, *SLC7A11*, *EMC2*, *G6PD*, and *ACSF2*) could serve as a novel biomarker for predicting BRCA patient’s prognosis [4]. Zhang et al. constructed a seventeen genes model as potential prognostic biomarkers for BRCA using multivariate Cox analysis and artificial intelligence-based algorithms [5]. The 6-lncRNA risk model and nomogram constructed have independent predictive ability for BRCA patients in the research of Luo et al. [6]. Bo et al. screened *LINC00467*, a key gene associated with oxidative lipid metabolism and immune infiltration, and could serve as a biomarker for diagnosis, metastasis, and recurrence of BRCA [7]. Xu et al. constructed an angiogenesis-related seven genes prognostic risk signature consisting of *BTG1*, *IL18*, *PF4*, *RUNX1*, *SCG2*, *THY1*, and *TNFSF12* for BRCA patients [8]. However, related research is in its infancy, and does not meet the requirement of BRCA patients' prognosis prediction.

In the present study, we aimed to construct a polygenic risk scoring model via bioinformatics and machine learning algorithm based on public databases. We believe that this study can provide new insight and detailed and reliable data for future scientific research and clinical precision medicine of BRCA patients.

## 2 Methods

### 2.1 Data source

The RNA-sequencing expression data and corresponding BRCA patient clinical data were collected from the Cancer Genome Atlas (TCGA). This combined set served as a training cohort, including 1098 BRCA and 113 normal samples. GSE162228 was obtained from the Gene Expression Omnibus (GEO) databases and served as a validation cohort, including 134 BRCA samples. The protein expression of the hub gene detected by immunohistochemistry in tumor and normal tissue was explored from the Human Protein Atlas (HPA) database (https://www.proteinatlas.org/).

### 2.2 Robust differently expressed genes identification

The R package “limma” was performed to normalize the RNA-seq expression data for eliminating the batch effect and ascertain the robust the differently expressed genes (DEGs) between breast tumor tissues and normal tissues with screening conditions including P < 0.01 and |log2FC| > 1 in the training cohort. The results were visualized by the ggplot2 R software package to draw a volcano plot, and the top 50 up-regulated and down-regulated genes were used as heat maps respectively.

### 2.3 Co-expression modules construction by weighted gene co-expression network analysis

The weighted gene co-expression network analysis (WGCNA), a method capable of transforming gene expression data into co-expression gene modules and exploring the relationship between modules and clinical features, was performed to construct co-expression modules using the expression data of DEGs. The batch effect of the data has been eliminated by the “removeBatchEffect” function of the package “limma” of R software. Then, using gene expression profiling, we calculated the median absolute deviation (MAD) of each gene separately, eliminated the top 50% of genes with the smallest MAD, and removed outlier genes and samples using the “good Samples Genes” method of the R package WGCNA. Scale independence and average connectivity degree of network with different power values were tested (ranging from 1 to 18). The appropriate power value was determined when scale independence was above 0.85 with a relatively higher connectivity degree. Then, according to topological overlap matrix (TOM)-based dissimilarities, genes were sorted into different gene modules. The “Pearson” method was performed to identify key gene modules according to a correlation between the module and clinical traits.

### 2.4 Construction of gene signature by the least absolute shrinkage and selection operator penalized Cox regression analysis

In our work, the least absolute shrinkage and selection operator penalized Cox (LASSO-Cox) was used to construct a prognostic model for minimizing the risk of overfitting using the function “glmnet” of the R package. Then, the risk score of patients was calculated based on gene expression and the corresponding Cox regression coefficient as follows: Risk score = β1 × mRNA 1EXP+β2 × mRNA 2EXP + …... + βn × mRNA nEXP, where β is the multivariate regression coefficient of the corresponding mRNA, and mRNA EXP is the expression level of the corresponding mRNA [9,10]. Patients were divided into high- and low-risk score groups according to the median risk score.

### 2.5 Survival analysis

The Kaplan–Meier method was used to analyze the relationship between risk score and the survival of patients with BRCA in training and validation cohort of our study. Subsequently, receiver operating characteristic (ROC) curves were used to evaluate the prediction ability of risk score on the survival probability by using the survival ROC package in R. Moreover, the COX regression analysis was used for calculating the association between clinical features and patient survival, and the forest plot was constructed using the R package “forest plot” to exhibit the hazard ratio (HR), 95% CI, and p-value. Last, the R package “rms” was utilized to build the nomogram. Moreover, survival analyses of hub genes were preformed via kmplot (https://kmplot.com) which is the more authoritative online analysis website for survival analysis over the world. The miRNAs of hub genes were be collected from the online website (http://mirwalk.umm.uni-heidelberg.de/search_genes/). Then, their common miRNA was screened to further carry out survival analysis.

### 2.6 Gene set enrichment analysis

The Gene Set Enrichment Analysis (GSEA) was the first to rate an ordered list of DEGs in this work. The GSEA was carried out to analyze the significant survival difference observed between the high-risk score group and the low-risk score group of patients with BRCA. The dataset of c2.cp.kegg.v7.4.symbols.gmt was downloaded from the molecular signatures database and was used to evaluate related pathways and molecular mechanisms. Set permutations were performed 1000 times for each analysis. P value of <0.05 and an FDR of <0.25 were considered statistically significant.

### 2.7 Analysis of immune cell infiltration levels

To research the relationship between the expression of the hub gene and six kinds of immune cells, we conducted the analysis of immune cell infiltration levels by the Tumor Immune Estimation Resource (TIMER) methods based on the TIMER2.0 database (http://timer.cistrome.org/) which was widely used to study immune cell infiltrates across various different tumors [11]. The related immune cells included B cell, CD8+T cell, CD4+T cell, macrophage, neutrophil, and dendritic cell, and the relationship was corrected according to tumor purity. Moreover, the BRCA patient was grouped into two groups by the expression level of the hub gene to research further correlations between the hub gene and the immune microenvironment score of six kinds of immune cells.

### 2.8 Protein-protein interaction network and functional enrichment analysis

To investigate the interaction between the hub genes and other genes, the Search Tool for the Retrieval of Interacting Genes/Proteins (STRING) website (https://string-db.org/) was used to research the related genes with a minimum confidence score >0.4. The online website hosts a big collection of integrated and consolidated protein-protein interaction data [12]. The Protein-protein interaction (PPI) network was constructed via the software of Cytoscape3.7.1.

Moreover, those genes were carried out further enrichment analysis. Then, the “clusterProfiler” R package 15 was employed to perform Kyoto Encyclopedia of Genes and Genomes (KEGG) analyses on the basis of those genes. P-values were adjusted by the Bebjamini and Hochberg method. The Gene Ontology (GO) function enrichment analysis was performed on the first 100 co-expressed genes, and cellular component (CC), biological process (BP) and molecular function (MF) were annotated.

### 2.9 Statistical analysis

The qualitative data between groups were compared with a rank-sum test using IBM SPSS 21.0 software (IBM SPSS, Armonk, NY, USA) as the data did not conform to a normal distribution. And the bilateral p values < 0.05 were considered statistically significant.

## 3 Results

**Fig. 1:**
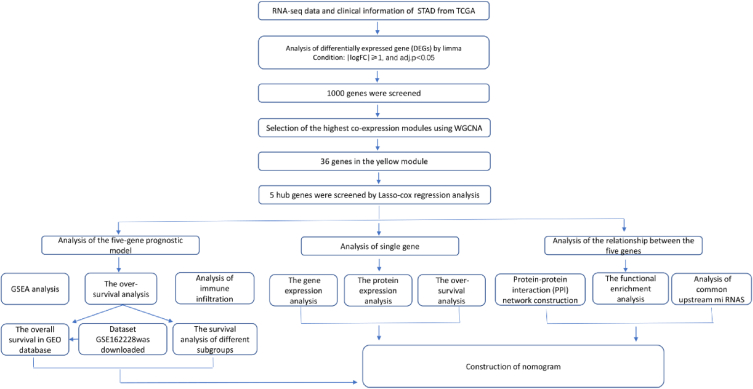
Flowchart of the data collection, processing, analyzing, validation by bioinformatics technology.

The flowchart of the study was shown in Fig. 1.

### 3.1 Identification of DEGs in TCGA-BRCA

1000 DEGs were found between the normal and tumor groups with the “limma” R package (adj. P value < 0.05 and |log2FC| > 1). The DEGs included 396 upregulated genes and 604 downregulated genes. And the DEGs were plotted into a volcano map (Fig. S1A). Moreover, the top 50 upregulated and downregulated DEGs were visualized as heatmaps (Fig. S1B).

### 3.2 Co-expression network construction and key module identification

**Fig. 2:**
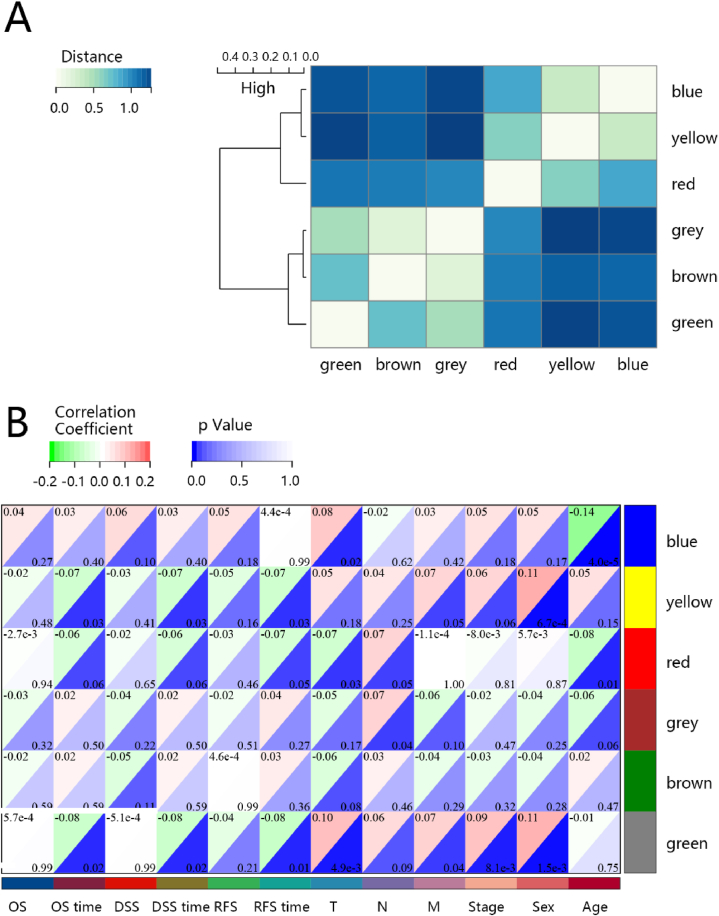
Identification of significant modules in WGCNA network. (A) Heatmap for the distance of module eigengenes each other. (B) Heatmap for the correlation between module eigengenes and clinical traits. OS is an abbreviation for over survival. DSS is an abbreviation for disease specific survival. RFS is an abbreviation for progression free survival.

The WGCNA was performed on the extracted DEGs to explore the correlation of gene modules with clinical features of interest. The clinical information of BRCA patients were retrieved from TCGA database. The results revealed that after setting a soft-thresholding value β as 9 and cut height as 0.20, the constructed gene co-expression network approximated a scale-free topology distribution with fitting R^2^ = 0.86 (Figs. S2A and S2B). In generally, the optimal soft threshold β is set as the minimum soft threshold when the fitting coefficient R^2^ approaches or reaches 0.9. A gene cluster tree was presented in Fig. S2C and six co-expression modules were eventually identified. And heatmap for the distance of module eigengenes each other was showed in Fig. 2A when cut height as 0.20. According to the heatmap of module-trait relationships (Fig. 2B), the yellow module demonstrates the highest correlations with clinical traits.

### 3.3 Construction of the five-gene prognostic model

**Fig. 3:**
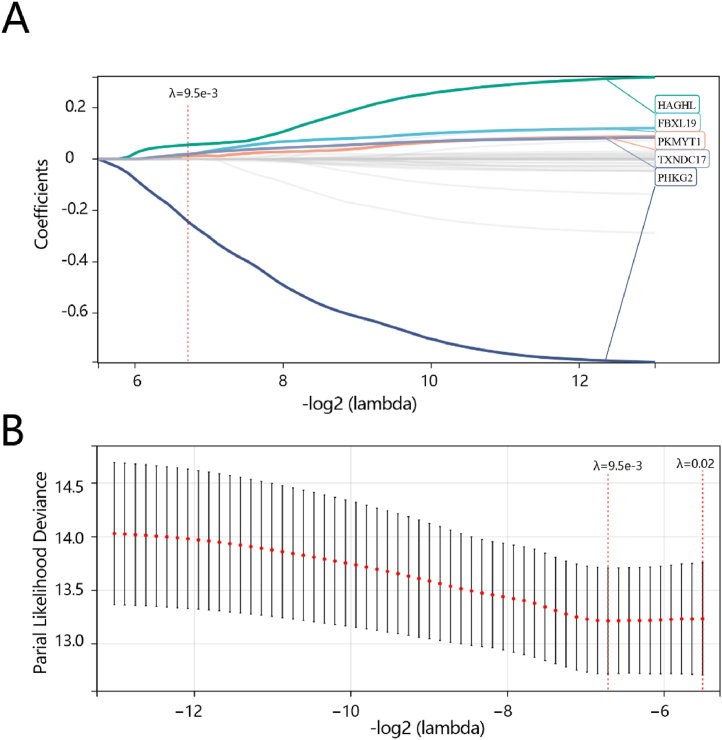
Hub genes were screened by LASSO analysis. (A) Selection of the optimal hub gene in the Lasso model (λ). (B) A coefficient distribution map for a logarithmic (λ) sequence was generated as appropriate.

According to the integrated survival time, survival status, and gene expression data, the Lasso-cox regression analysis was performed on 36 hub genes within the yellow module. And 5 biomarkers: *FBXL19*, *HAGHL*, *PHKG2*, *PKMYT1*, and *TXNDC17* (Fig. 3A) were screened when the tuning parameter (λ) was minimized to 9.5e-3 and log (λ) was 0.02 (Fig. 3B). Finally, a five-gene prognostic model was constructed to predict prognosis with the following equation, risk score = 0.0151764285738641*the expression of *FBXL19* + 0.0553017583288283*the expression of *HAGHL*-0.244035030263264*the expression of *PHKG*2 + 0.0111079001204152*the expression of *PKMYT1*+0.0191186055187776*the expression of *TXNDC17*. According to the median value of the risk score, the patients were divided into a high-risk group or a low-risk group in the TCGA cohort.

### 3.4 Evaluation and validation of the five-gene signature

**Fig. 4:**
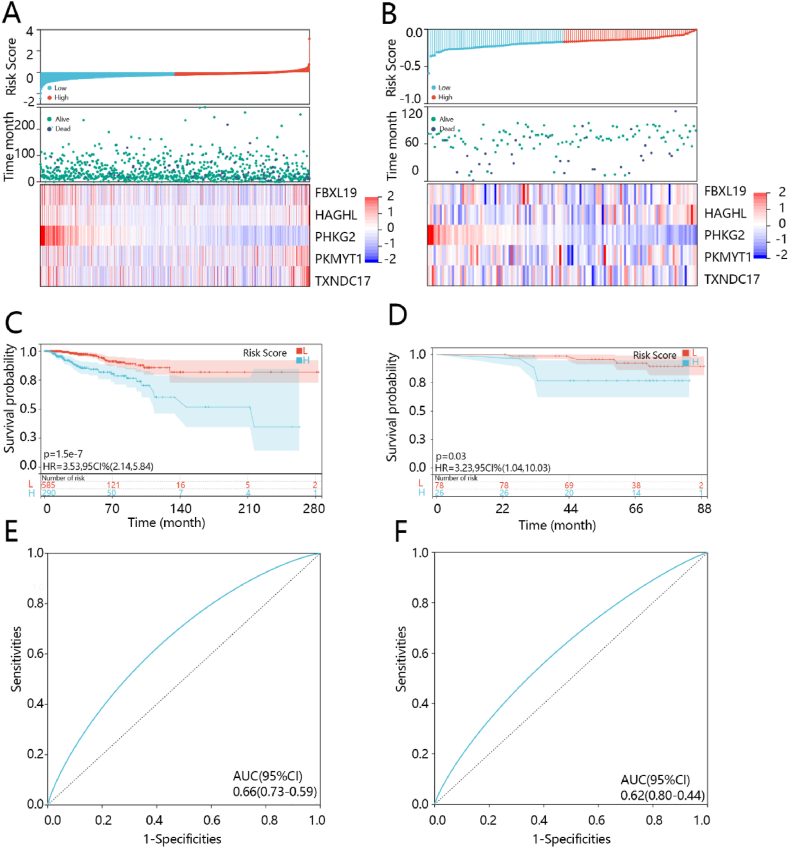
The performance of the five-gene model in training (the left side of the figure) and verification (the right side of the figure) cohort, separately. (A–B) The Z correction value of risk score distribution, the risk score density distribution, and the five gene expression heat maps. (C–D) Survival analysis. (E–F) ROC curve.

The relationship between prognostic models and gene expression in models for training cohort and validation cohort were shown in Fig. 4A and B, respectively. Survival analysis showed that risk score significantly affected the prognosis of BRCA patients (P < 0.001), and the survival rate of patients in the high-risk score group was low (Fig. 4C). The area under the curve (AUC) for the ROC analysis was 0.66 (Fig. 4E), indicating good predictive capability of the model. The prognostic model was further verified in the GSE162228 dataset. Analogously, the result showed that the survival rate of patients in the high-risk score group was low (Fig. 4D) with a dramatic difference, and AUC for the ROC analysis was 0.62 (Fig. 4F). These results commonly suggested the favorable prediction performance of established prognostic model.

### 3.5 Cox regression analysis of the five-gene signature

**Fig. 5:**
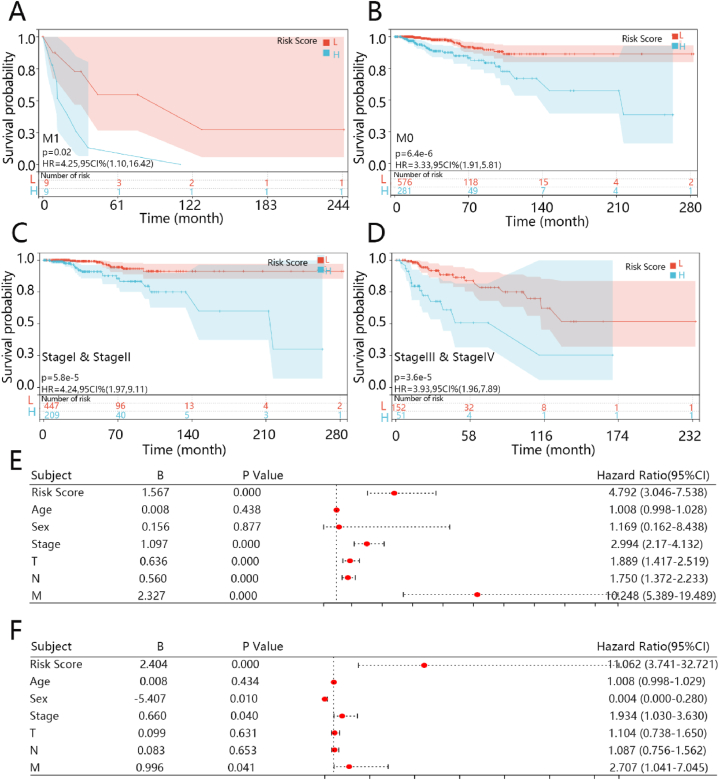
Analysis of univariable and multivariable cox regression of five-gene signature and survival analysis of subgroups. (A–D) Survival analysis of different subgroups assessing the independence of risk score. (E) Forest plots of univariable cox model. (F) Forest plot of the multivariable cox model. The hazard ratio (HR) is a relative prognostic measure of patients with breast cancer.

To prove the prognostic value of the five-gene signature in BRCA patients, subgroup survival analysis on risk score was conducted regarding the independent prognostic factors of M stage and clinical stage. All samples were classified into different cohorts according to the status of M stage and clinical stage, and Kaplan-Meier analysis was used to evaluate the prognostic value of risk score in BRCA. The results showed that there were still significant differences between the two groups (P < 0.05), and the five-gene signature distinguished the high-risk score group from the low-risk score group (Fig. 5A–D) when confounding factors of M and stage were eliminated.

Subsequently, the Cox regression analyses were performed on the five-gene risk score, age; sex, clinical stage, and pathological tumor, node, and metastasis (T, N, M) stage. The five-gene risk score, T, N, M stage, and clinical stage were significantly associated with prognosis in the univariable analyses (Fig. 5E; P < 0.05) and there was no significant difference in age and sex. In the multivariable analyses, the five-gene risk score, sex, M stage, and clinical stage can independently predict the patient’s prognosis (Fig. 5F; P < 0.05).

### 3.6 Protein-protein interaction network analysis and genes enrichment analysis

**Fig. 6:**
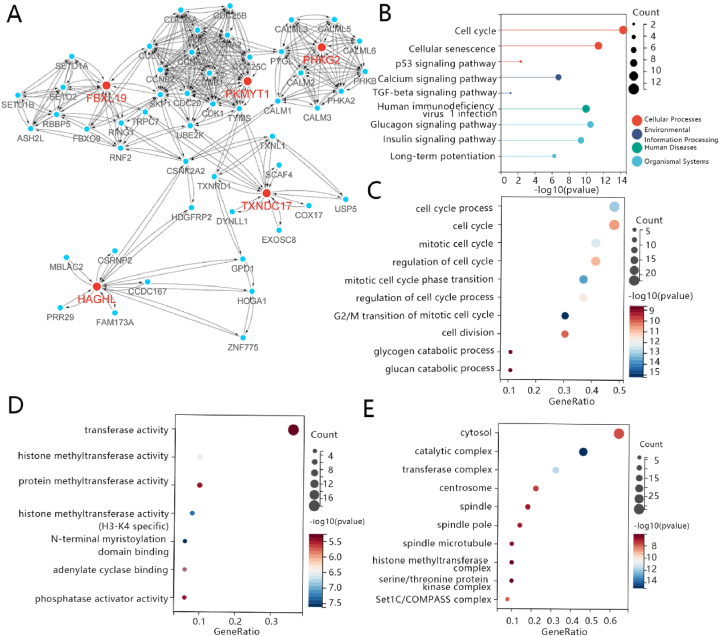
Construction of protein-protein interaction network and related genes enrichment analysis. (A) The determination of 5 hub genes within the whole PPI network. Red dot represents hub genes, and blue dot represents related genes (B) KEGG Enrichment plots of 5 hub genes and their related genes. (C) Go enrichment analysis of 5 hub genes and their related genes in BRCA molecular function, (D) biological process, and (E) cellular component. The abscissa gene ratio indicates the number of differential genes located in the gene databases/the total number of genes located in the gene databases. Gene ratio is positively correlated with enrichment degree. The ordinate is description information for pathway. The size of the dot represents the number of genes with significant difference that S gene number matches to a single pathway, and the color of the dot represents the P value of enrichment analysis. (For interpretation of the references to color in this figure legend, the reader is referred to the Web version of this article.)

The PPI network was established based on key genes and other related genes. These related genes were collected from the website of string. The PPI network included 54 points and 386 edges. The PPI network showed that some proteins had a close relationship with hub proteins, for example, RNF2 UBE2K, TXNL1, CALM1, and TRPC7(Fig. 6A). KEGG enrichment and GO enrichment analyses were conducted based on the genes that coded for related proteins. The results of KEGG enrichment analysis should that those genes were enriched in cell cycle, cellular senescence, p53 signaling pathway, calcium signaling pathway, TGF-beta signaling pathway, human immunodeficiency virus 1infection, Glucagon signaling pathway, insulin signaling pathway, and long-term potentiation (Fig. 6B). The result of Go enrichment analysis showed that MF mainly includes cell cycle process, cell cycle, mitotic cell cycle, regulation of cell cycle, mitotic cell cycle phase transition, regulation of cell cycle process G2/M transition of mitotic cell cycle, cell division, glycogen catabolic process, glucan catabolic process (Fig. 6C); BP mainly includes transferase activity, histone methyltransferase activity, protein methyltransferase activity, histone methyltransferase activity (H3-K4 specific), N-terminal myristoylation domain binding, adenylate cyclase binding, and phosphatase activator activity (Fig. 6D); CC mainly includes cytosol, catalytic complex, transferase complex, centrosome, spindle, spindle pole, spindle microtubule, histone methyltransferase complex, serine/threonine protein kinase complex. (Fig. 6E).

### 3.7 Analysis of immune cell infiltration levels

The TIMER database was used to explore the immunological microenvironment and identified correlations between levels of immune infiltration and expressions of the hub genes in BRCA (Fig. S3A). The results showed that the expression of different genes correlated differently with different immune cells. For instance, the results showed that *FBXL19* expression had correlation with B cell infiltration (r = −0.169, p = 6.03e−02), and CD8+T cell (r = −0.174, p = 5.44e−02); *HAGHL* expression had correlation with B cell infiltration (r = −0.13, p = 1.52e−01), and Macrophage (r = −0.323, p = 2.14e−04); *PHKG2* expression had correlation with CD8+T cell (r = 0.102, p = 2.62e−01), and Neutrophil (r = 0.12, p = 2.12e−01); *PKMYT1* expression had correlation with CD8^+^ T Cell (r = −0.176, p = 5.21e−02), Macrophage (r = −0.256, p = 3.72e−03); *TXNDC17* expression had correlation with CD4^+^ T Cell (r = −0.224, p = 1.31e−02), Neutrophil (r = −0.222, p = 1.98e−02). Moreover, the immune microenvironment score of the macrophage in the low-risk score group was significantly lower than in the high-risk score group (Fig. S3B).

### 3.8 GSEA

**Fig. 7:**
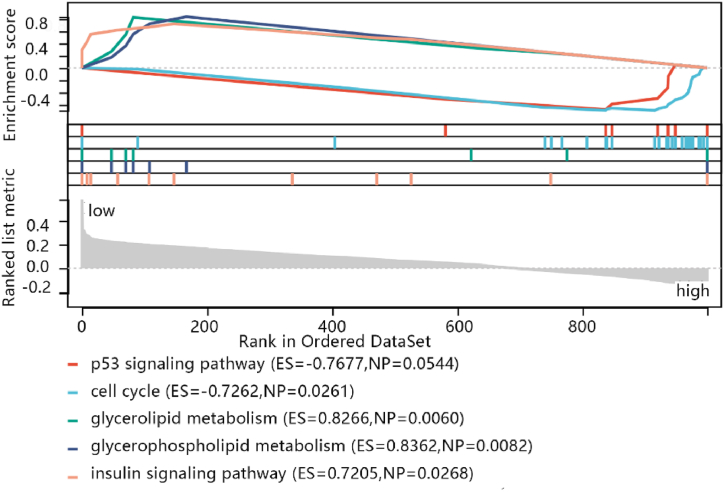
Enrichment plots from set enrichment analysis (GSEA). The different lines represent different pathways. ES is an abbreviation for enrichment score, which is the degree of enrichment of reflective gene set members S at both ends of the sorted list L. NP is an abbreviation for nominal p value.

The results of the GSEA enrichment analysis showed that two pathways including P53 signaling pathway (ES = −0.7677, NP = 0.0544) and cell cycle (ES = −0.7262, NP = 0.0261) were associated with the high-risk score group. There were three pathways were associated with the low-risk score group. Those pathways contained glycerolipid metabolism (ES = 0.8266, NP = 0.0060), glycerophospholipid metabolism (ES = 0.8362, NP = 0.0082), insulin signaling pathway (ES = 0.7205, NP = 0.0268) (Fig. 7).

### 3.9 Construction of nomogram

**Fig. 8:**
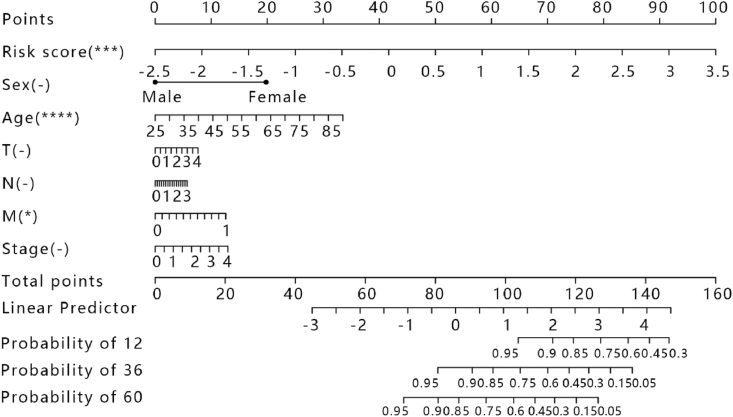
The nomogram for predicting the proportion of patients with 12-month overall survival, 36-month overall survival, and 60-month overall survival.

We assessed the 1,3,5-years survival probability by constructing the nomogram that could integrate various clinical prognostic factors to predict survival. Thus, a risk prediction nomogram integrating the five-gene signature, age, stage, TNM stage, and sex was constructed (Fig. 8). The C-index, hazard ratio, and the P value of this model were 0.754, 95%CI (0.698–0.809), and 2.106e−19, separately. The result showed that the model had good work in determining the patient’s disease progression and was able to make a personalized prediction of the BRCA patient.

### 3.10 Validation of the expression of the five-gene signature

**Fig. 9:**
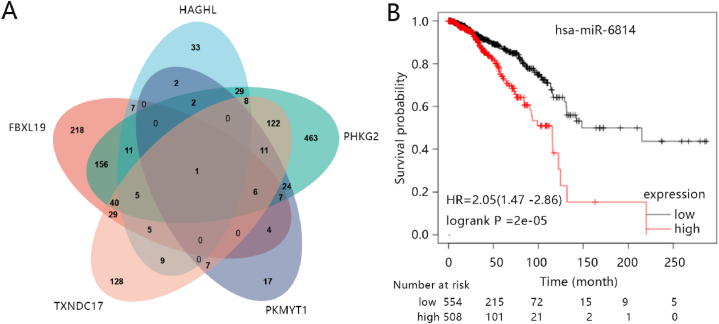
Analysis of the miRNA of hub genes. (A) The Venn plot of miRNA related hub genes. (B) The survival analysis of common miRNA for 5 hub genes. The hazard ratio (HR) is a relative prognostic measure of patients with BRCA. p was used to determine the level of prognostic significance of patients with BRCA. Furthermore, the p < 0.05 was meant as a significant difference in the prognostic expression of patients with BRCA.

To validate the clinical value of a five-gene signature, we explored five genes expression in BRCA. The result showed all genes were significantly upregulated in tumors in TCGA-BRCA all samples (Fig. S4A; p < 0.001). We analyzed the protein expression levels of the five genes using the HPA database. The result showed that *HAGAL* and *TXNDC17* were lowly expressed in cancer (Figs. S4B and S4C). The expression of *FBXL19*, *PHKG2*, and *PKMYT1* were not recorded in the HPA database. Furthermore, survival analysis showed that low expressions of *FBXL19*, *HAGHL*, and *TXNDC17* were significantly correlated with longer over survival (Fig. S5A, S5C and S5E; P < 0.05); and high expression of *PHKG2* was significantly correlated with longer over survival time (Fig. S5D; P < 0.05); and expression of *PKMYT1* was irrelevant to over survival in the TCGA-BRCA dataset (Fig. S5B). Moreover, we screened the miRNA related with 5 hub genes from the website named http://mirwalk.umm.uni-heidelberg.de/search_genes/. Then, the overlapped miRNA was screeded by a Venn plot and named has-miR-6814 (Fig. 9A). The survival analysis showed that low expressions of has-miR-6814 were significantly correlated with longer over survival time (Fig. 9B).

## 4 Discussion

BRCA is the most common interstitial breast disease in the world female. In addition, it is a highly heterogeneous malignant disease with a great change and the worst prognosis. The survival of individual patients is difficult to predict [13]. Therefore, screening suitable prognostic markers is an urgent need for precision medicine of BRCA patients.

This paper focused on the analysis of DEGs whose expression was different with significance between the breast normal tissue and tumor tissue, and determined 5 genes (*FBXL19*, *HAGHL*, *PHKG2*, *PKMYT1*, and *TXNDC17*) as prognostic biomarkers for BRCA by WGCNA and Lasso-cox methods. Based on 5 genes, firstly, we constructed a risk score model to predict the survival of BRCA patients, and the model showed that the low-risk score group was beneficial for patient prognosis. Then, the model was verified in the GEO database. Secondly, a risk prediction nomogram integrating the five-gene signature, age, stage, TNM stage, and sex was constructed to provide more precise mortality risk prediction and better treatment options for BRCA patients. Lastly, the expression levels of the above genes in BRCA were further verified by TCGA-BRCA, and it was found that the expressions of *FBXL19*, *HAGHL*, *PHKG2*, *PKMYT1*, *TXNDC17* in BRCA tissues were significantly higher than those in adjacent tissues. But the protein expression levels of *HAGAL* and *TXNDC17* were lowly expressed in BRCA in the HPA database. And the expression of *FBXL19*, *PHKG2*, and *PKMYT1* were not recorded in the HPA database.

*FBXL19*, F-Box and Leucine Rich Repeat Protein 19, encodes a member of the Skp1-Cullin-F-box family of E3 ubiquitin ligases. The reported researches on *FBXL19* and *HAGHL* were rare. Dong et al. uncovered that *FBXL19* was a tumor suppressor in esophageal cancer, by regulating the degradation and ubiquitination of Rac3 protein which plays an important role in the progress of esophageal cancer cells, and then inhibiting the downregulation of cadherin induced by TGF Bata1 pathway [14]. In this work, we found that the expression of *FBXL19* gene was higher in breast tumor tissue than in normal tissue in the TCGA database, and low expressions of *FBXL19* was significantly correlated with longed over survival time. it may be a BRCA-promoting gene by regulating different pathways. The function of *FBXL19* in BRCA is valuable and needs further study. *HAGHL*, Hydroxy acyl glutathione Hydrolase-Like Protein, is an important paralog of *HAGH*. Im et al. indicated that *HAGHL*, related to bone resorption pathways, was a significant genetic marker of fracture risk in female children based on the Childhood Cancer Survivor Study database [15]. *PHKG2*, phosphorylase kinase, belongs to the phosphorylase b kinase (PhK) subunit, containing the active site of the enzyme, and it is related to the activation of cAMP-dependent protein kinase A and the glycogen metabolism pathway. Moreover, it encodes the hepatic isoform of the gamma unit of phosphorylase kinase. People get glycogen storage disease type IXc, a rare and severe phenotype of glycogen storage disease, when *PHKG2* is defective [16,17]. The reported research showed that *PHKG2* was frequent epigenetic hypermethylation in papillary thyroid cancer [18]. And Fu et al. identified that the mRNA expression level for the *PHKG2* was elevated in BRCA cell lines [19]. Consistently, the result of our verification of *PHKG2* expression in TCGA-BRCA that its expression in BRCA was higher than in adjacent tissues. Higher expression of *PHKG2* may disrupt functionally important and BRCA-related genes [20]. In lung adenocarcinoma, *PHKG2* is involved in the process of ferroptosis which prevents tumor development [21,22]. *PHKG2* positively regulates ferroptosis through the modulation of available iron, silencing may function as iron chelation [23,24]. It is possible that it is also involved in the ferroptosis process and inhibits tumor development in BRCA.

*PKMYT1*, protein kinase, membrane associated tyrosine/threonine 1, is a member of the serine/threonine protein kinase family, and is reported to relate to negatively regulates the G2/M transition of the cell cycle and assemble Golgi and endoplasmic reticulum by phosphorylating and inactivating cyclin-dependent kinase 1 [25]. Liu et al. study showed that the *PKMYT1* regulates EMT by activating beta-catenin/TCF signaling in the hepatocellular tumor cell [26]. In esophageal squamous cell carcinoma, *PKMYT1* promotes cellular proliferation, migration and infiltration through activation of the AKT/mTOR signaling pathway [27]. Toledo et al. study showed that the knockout of *PKMYT1* significantly causes cell death and stalls the cell cycle [28] because that it is a cell cycle-regulated kinase that inhibited Cdc2 activity by phosphorylating Cdc2 at Thr14 and Tyr15. Liu et al. study showed that the high expression of *PKMYT1* was related to poor prognosis and the ER-, PR-hormone levels of BRCA patients [29]. In this work, the high expression of *PKMYT1* was harmful to the patient’s prognosis.

*TXNDC17* (also named thioredoxin⁃related protein 14 (*TRP14*), thioredoxin-like 5 (*TXNL5*)) is disulfide reductase and belongs to the thioredoxin family [30]. The research closely interrelated to *TXNDC17* in cancer was rare. A few of researches shown that *TXNDC17* promoted the growth and development of cancer cells by regulating the autophagy signal pathway. For instance, the reported research showed that *TXNDC17* promoted paclitaxel resistance via inducing autophagy in ovarian cancer [31]. It also played a key role in cisplatin resistance through the induction of autophagy [32]. The finding of this work showed that the expression of *TXNDC17* in BRCA tissue was higher than in the normal breast tissue, and the its high expression had a poor prognosis.

Tumor builds upon continuous proliferation of cell due to cell cycle abnormal [33]. In this research, results of the GSEA analysis showed that the high-risk score group enriched the P53 signaling pathway and cell cycle and suggested that hub genes affected the development of tumors through these signaling pathways. Consistently, PPI showed that hub genes had interacted with the cell cycle-related protein, for instance, CDK1, and CDC20. Immune cell infiltration affects the clinical outcomes of patients with cancer [34]. Both in physiological and pathological angiogenesis, macrophages are thought to be an important bridge to angiogenesis [35]. The immune microenvironment score of the macrophage in the low-risk score group was significantly lower than in the high-risk score group in this study. There was a reason that macrophage promotes angiogenesis, which in turn promotes tumor development via secreting vascular endothelial growth factors and IL-12 [36].

In discovery, the nomogram that was constructed by combining five gene signatures, clinical stage, age, TNM stage, and sex had a good prediction accuracy for 1,3,5-years survival times and can be used as a practical and reliable prognostic tool for BRCA patients. In this work, the finding showed that protein expression levels of *HAGAL* and *TXNDC17* were lowly expressed in BRCA cancer using the HPA database, but expression levels of *HAGAL* and *TXNDC17* were high. There are two hypothetical reasons for explaining the situation that mRNA abundance is inconsistent with protein expression levels. On one hand, the process of post-transcriptional regulation, translation, and post-translational regulation that all play a role in the expression level of the final protein was affected; on the other hand, the protein was degraded. However, the specific mechanism needs to be further explored.

Although our five genes' risk model and nomogram provide new guidance for prognosis of BRCA patients, the study still has some limitations. Firstly, the TCGA database is biased due to only containing US patients. Second, there was no study experiment to explore the function of five genes in BRCA-related signaling pathways due to the availability of clinical specimens and experimental conditions. Therefore, the specific mechanisms of how five genes affect the prognosis of BRCA patients require further attention and research.

## 5 Conclusions

In summary, the 5 genes risk model constructed in this study has an independent predictive ability to distinguish patients with a high risk of death from those with a low-risk score in both derivation and validation cohorts, which should provide an option for predicting prognosis in BRCA patients. And the nomogram established is helpful for the individualized survival prediction of BRCA patients. Further investigation is needed in future research.

## Author contribution statement

Du XJ, and Yang XR conceived and designed the experiments, and analyzed and interpreted the data. Wang QC and Lin GL contributed reagents, materials, analysis tools, and data; Li PF wrote the paper; Zhang WF analyzed and interpreted the data in revising the manuscript.

## Funding statement

This research did not receive any specific grant from funding agencies in the public, commercial, or not-for-profit sectors.

## Data availability statement

Data will be made available on request.

## Declaration of interest’s statement

The authors declare no conflict of interest.
