# Supplementary material for: Identification and validation of a five-gene prognostic signature based on bioinformatics analyses in breast cancer
Source: Heliyon. 2023 Jan 27;9(2):e13185. doi: 10.1016/j.heliyon.2023.e13185 (PMC9898304; doi:10.1016/j.heliyon.2023.e13185)
Supplement: Multimedia component 1 [file mmc1.docx]

Supplementary


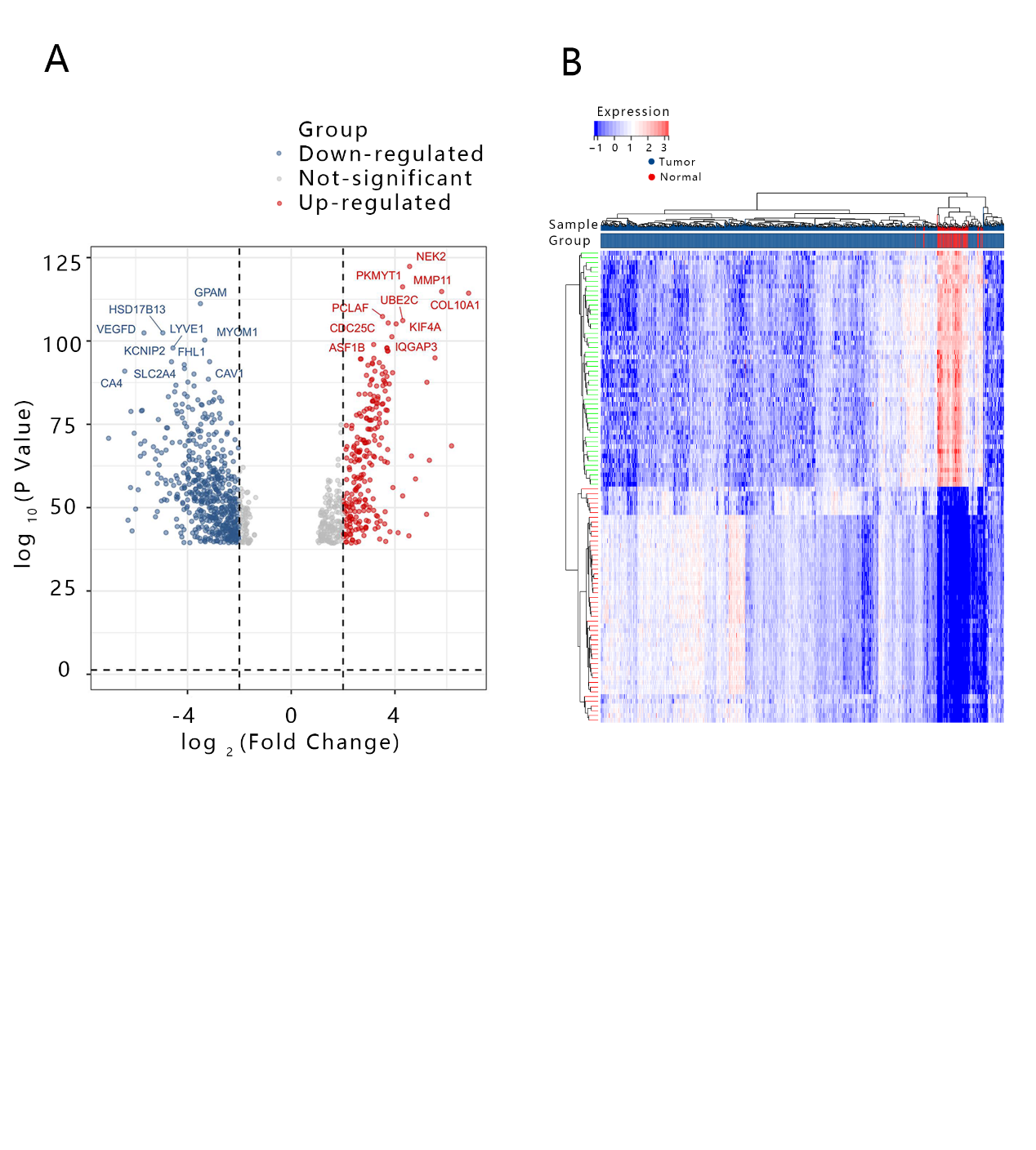


**Figure S1.** Identification of differentially expressed genes. **(A)** Volcano map of differential genes, different points represent different RNAs. The upregulated genes in the samples are in red, while the downregulated are in blue. **(B)** Heat map of differentially expressed genes. The upregulated genes in the samples are in red, while the downregulated are in blue. The expression of genes in the tumor group and the normal group were quantified into two obvious panels.


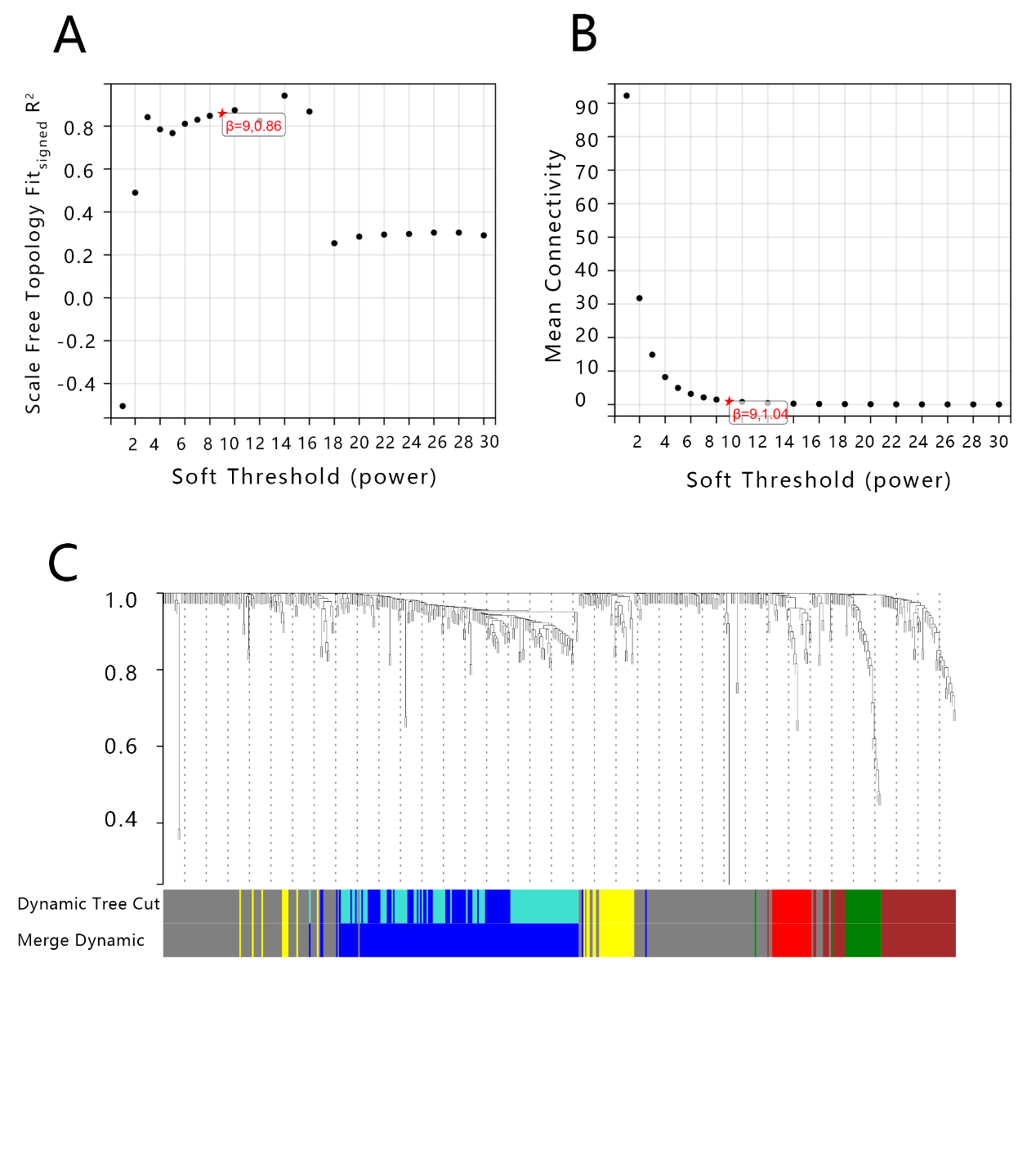


**Figure S2.** Construction of gene modules based on DGEs through WGCNA. **(A)** Analysis of the scale-free fit index for various soft-thresholding powers (β=9). **(B)** The mean connectivity for various soft-thresholding powers. **(C)** Dendrogram of genes clustered based on the dissimilarity measure. Different colors represent different gene modules.

**
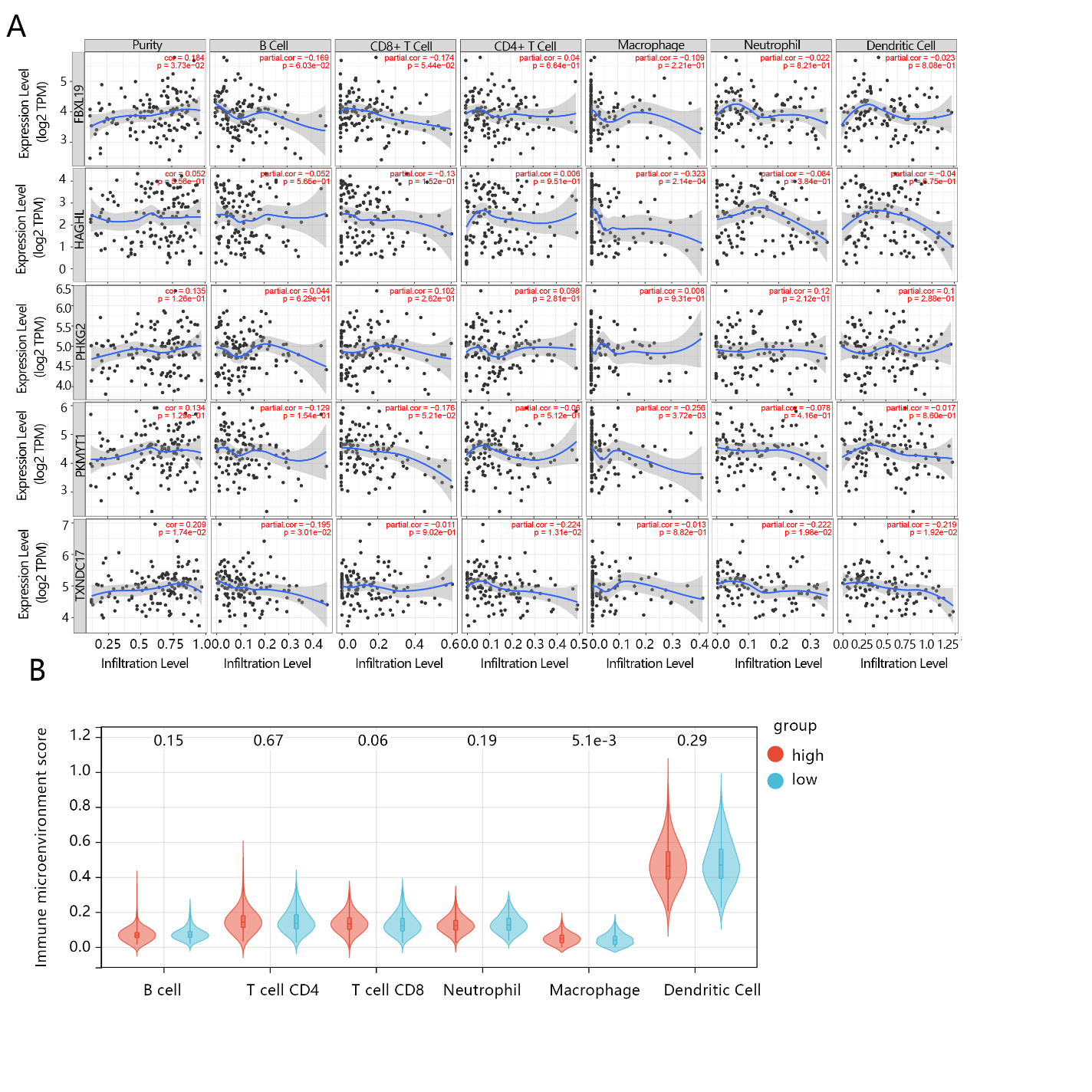
**

**Figure S3** Analysis of immune infiltration. (A) Correlation scatter plot of hub genes and six kinds of immune infiltration cells in BRCA. The abscissa represents the infiltration level of six types of immune cells. The ordinate represents the expression level of five hub genes. (B) The difference in the infiltration of different immune cells in high risk score group and low risk score group. The abscissa represents the six types of immune cells.


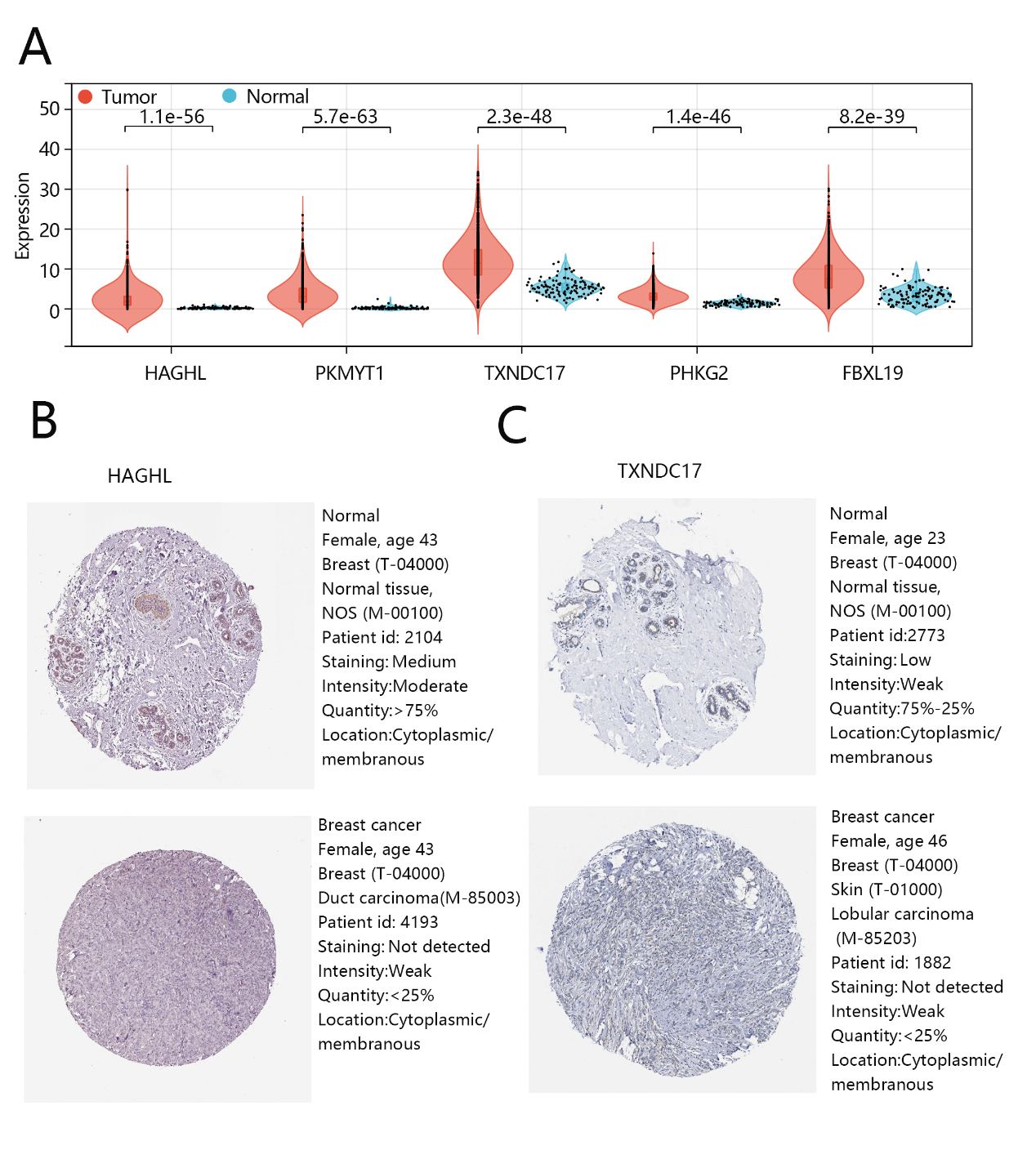


**Figure S4.** The mRNA and protein expression of five genes in breast tumor and normal samples. **(A)** Comparison expression of five-gene signature between BRCA tissue and normal samples. **(B)** Expression of HAGAL in the Human Protein Atlas protein database. **(C)** Expression of in TXNDC17 the Human Protein Atlas protein database.


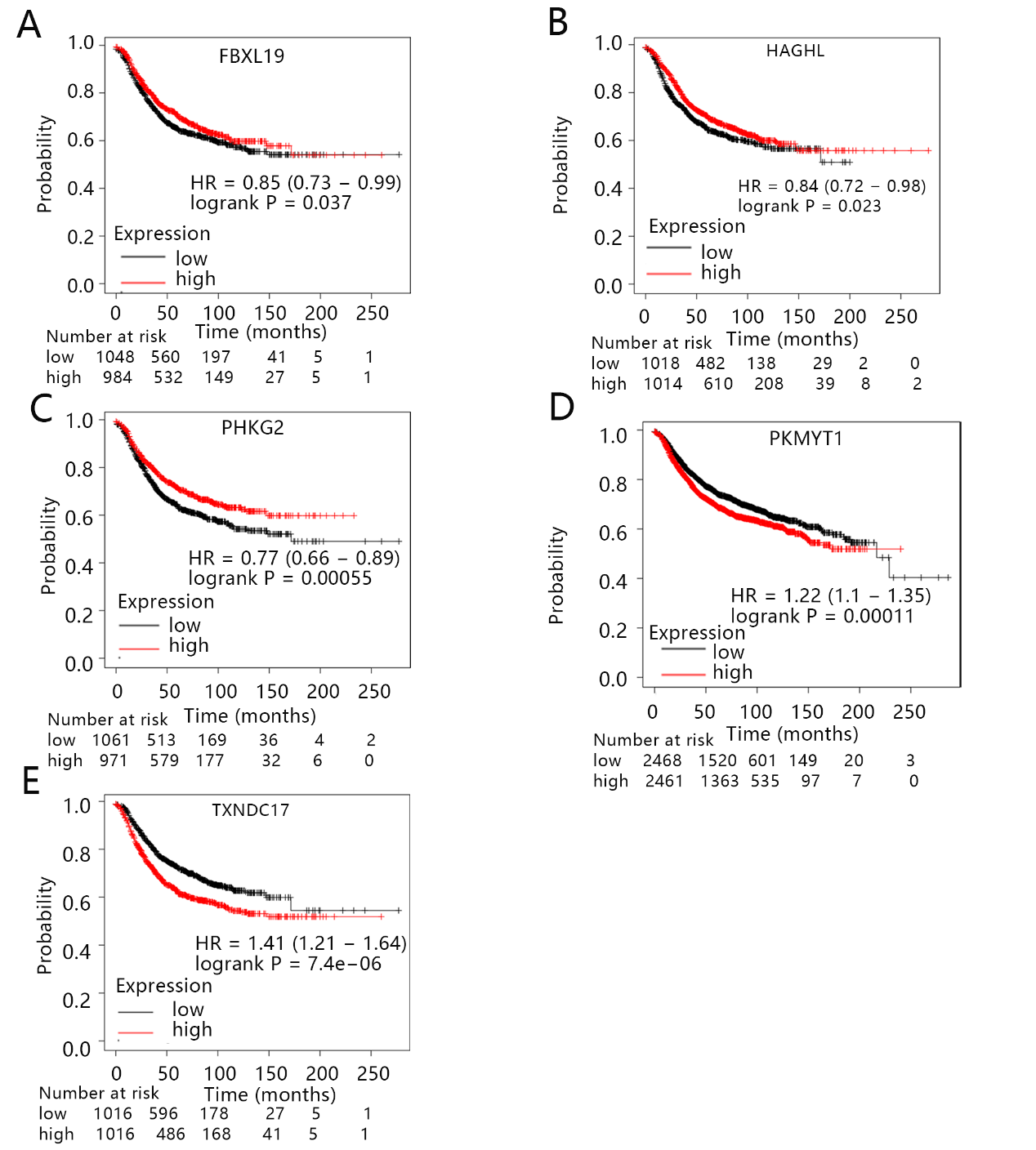


**Figure S5** Kaplan-Meier curves were plot for five genes expression via the online tool of kmplot. (A) *FBXL19*. (B) *HAGHL*. (C) *PHKG2*. (D) *PKMYT1*. (E) *TXNDC17*. The hazard ratio (HR) is a relative prognostic measure of patients with BRCA. p was used to determine the level of prognostic significance of patients with BRCA. Furthermore, the p < 0.05 was meant as a significant difference in the prognostic expression of patients with BRCA.
